# Challenges in Functional Food Products with the Incorporation of Some Microalgae

**DOI:** 10.3390/foods13050725

**Published:** 2024-02-27

**Authors:** Abuzer Çelekli, Buket Özbal, Hüseyin Bozkurt

**Affiliations:** 1 Department of Biology, Faculty of Art and Science, Gaziantep University, 27310 Gaziantep, Turkey; celekli.a@gmail.com (A.Ç.); buketozbal@gmail.com (B.Ö.); 2 Department of Food Engineering, Faculty of Engineering, University of Gaziantep, 27310 Gaziantep, Turkey

**Keywords:** bioactive chemicals, functional food, microalgae, nutrition

## Abstract

Much attention has been given to the use of microalgae to produce functional foods that have valuable bioactive chemicals, including essential amino acids, polyunsaturated fatty acids, vitamins, carotenoids, fiber, and minerals. Microalgal biomasses are increasingly being used to improve the nutritional values of foods because of their unique nutrient compositions that are beneficial to human health. Their protein content and amino acid composition are the most important components. The microalgal biomass used in the therapeutic supplement industry is dominated by bio-compounds like astaxanthin, β-carotene, polyunsaturated fatty acids like eicosapentaenoic acid and docosahexaenoic acid, and polysaccharides such as β-glucan. The popularity of microalgal supplements is growing because of the health benefits of their bioactive substances. Moreover, some microalgae, such as *Dunaliella*, *Arthrospira* (*Spirulina*), *Chlorella*, and *Haematococcus*, are commonly used microalgal species in functional food production. The incorporation of microalgal biomass leads not only to enhanced nutritional value but also to improved sensory quality of food products without altering their cooking or textural characteristics. Microalgae, because of their eco-friendly potential, have emerged as one of the most promising and novel sources of new functional foods. This study reviews some recent and relevant works, as well as the current challenges for future research, using different methods of chemical modification in foods with the addition of a few commercial algae to allow their use in nutritional and sensory areas. It can be concluded that the production of functional foods through the use of microalgae in foods has become an important issue.

## 1. Introduction

This review reports commonly used microalgae in the food industry with their advantages, the challenges of microalgae use in the food industry, and recent applications of microalgae in the food industry. Therefore, this review is constructed of the following sections: functional food, microalgae and their valuable metabolites, functional food products with the incorporation of microalgae, new trends in microalgae food application, and challenges in food products with microalgae.

One of the sustainable development goals is to provide nutrient-rich food for ending world hunger and to feed the growing world population, a significant worldwide societal concern. Huge amounts of food will be needed to sustain a global population of 9.7 billion in 2050 [1]. The rate of urbanization will increase, and by 2050, approximately 70% of the world’s population will be predominantly urban [2]. Projections of global food demand and consumption have a significant impact on the predicted rise in food production and its accompanying effects on biodiversity, land use change, and climate change [2]. The increasing global population endangers food security and exacerbates strain on restricted global resources, which leads to a major problem in supplying protein and sustainable food production [3]. *Food Security and Nutrition in the Age of Climate Change* reported that two billion people worldwide suffer from malnutrition due to inadequate nutrient intake, such as protein, amino acids, and calorie consumption [4]. Global climate change, decreases in agricultural land use, freshwater shortage, and environmental pollution affect food production levels and human health. The ever-increasing population of the world is one of the primary drivers behind the ongoing search for sustainable, risk-free, and alternative nutrient sources to satisfy the ever-increasing demand for food.

Due to time constraints, the development of technology and urbanization and the preferences of consumers are evolving in the present day. Today, the market for food additives is continuously expanding and is projected to continue to expand in the future [5]. Due to the high levels of bioactive compounds utilized to improve food composition, microalgae have garnered significant interest [5,6,7].

### 1.1. Functional Food

Diet and health are two of the most important aspects of people’s lives, and they merge in the study of functional foods. It is widely acknowledged that the relationship between diet and disease is the cornerstone of preventative nutrition. “Functional foods” are frequently recognized as a newly developing area. However, this concept was originally detailed in ancient Indian Vedic literature and in traditional Chinese medicine. One of the most important ideas in eastern philosophy is that “medicine and food come from the same source”. This idea is reflected in the desire to make functional foods [8].

In the 1980s, in response to growing healthcare expenses, the determination to make functional foods initially arose in Japan. In order to ensure the safety of certain foods, the Ministry of Health and Welfare devised a regulatory system [9,10]. Its main aim is to improve the well-being of the country’s aging citizens. The Japanese Ministry of Education, Science, and Culture began a national examination into the link between food and medical science in 1984 [11]. The term “functional food” was coined by Swinbanks and O’Brien [12] in *Nature* under the headline ”Japan examines the boundary between food and medicine”. Food for Specified Health Use and “Food with Nutrient Function Claims” are two types of labeling that make health claims [10].

The functional food market is a mostly global market that is not recognized by legislation everywhere. The term “functional food” can be defined in a variety of ways. Foods are “identical in appearance for conventional foods, ingested as part of a normal diet, with demonstrated physiological advantages and/or to minimize the risk of chronic illness beyond fundamental nutritional functions” [13]. The International Food Information Council defines functional foods as “foods or dietary components that may deliver health advantages beyond basic nutrition” [14]. A food is deemed functional “if it is demonstrated to affect beneficially one or more target functions in the body, beyond adequate nutritional effects, in a manner that is relevant to improve the state of health and well-being and/or a reduction in disease risk”, according to the European Commission’s Concerted Action on Functional Food Science in Europe [15].

There are multiple ways in which foods can be considered functional: (i) as a natural food, (ii) as a food to which a component has been added or removed, (iii) as a food with one or more components that have been modified, (iv) as a food whose bioavailability has been altered, or (v) as some combination of these. Table 1 illustrates categories of functional foods obtained from the study of Arvanitoyannis and Van Houwelingen-Koukaliaroglou [16].

The desire for nutritious and useful foods has prompted the exploration of new food categories to supplement the typical diet and the discovery of more holistic approaches to disease prevention and treatment [10,17]. Microalgae have received remarkable attention for the use of their biomass to develop multifunctional food products that are beneficial to human health.

### 1.2. Microalgae and Their Valuable Metabolites

Microalgae are unicellular photosynthetic microorganisms capable of converting solar energy into biochemical energy [18] and biomass containing a variety of useful substances for health, food and feed additives, cosmetics, and energy generation [19,20]. Nutrients and other health benefits can be gained from consuming microalgal biomass as a dietary supplement. Microalgae contain nutrient-rich bioactive compounds such as protein, essential amino acids, sulfated polysaccharides, enzymes, fibers, lipids, carotenoids, and vitamins [19,20,21,22]. The primary components of microalgal biomass are summarized in Table 2. Highly bioavailable protein is one of the major substances found in microalgae. Most microalgae biomass can contain more than 50% protein as dry weight. 

Microalgae include an abundance of vitamins (e.g., A, C, niacin, B1, B2, B6, etc.) and minerals (e.g., magnesium, potassium, iodine, iron, and calcium). Due to their high amounts of essential nutrients (Table 2, Table 3 and Table 4), microalgal biomasses are an important food source, especially in Asian countries such as China, Japan, and Republic of Korea [35]. Asian nations have utilized green microalgae as a dietary supplement or food source for hundreds of years. They are currently consumed worldwide for their rich nutritional content [19,20,36]. 

A few green microalgae (e.g., *Dunaliella salina*, *Chlorella vulgaris*, and *Haematococcus pluvialis*) and some cyanobacteria (e.g., *Arthrospira platensis*, synonym of *Spirulina platensis*) are biotechnologically important. They can be used as nutritional supplements for human food and additives for animal feed [22]. This is why they are commercialized. *Arthrospira platensis* and *Chlorella vulgaris* have been highlighted as natural sources of protein, whereas *Dunaliella salina* and *Haematococcus pluvialis* are considered natural sources of pigment (especially because of their content of β-carotene and astaxanthin, respectively) [19,20,22]. 

*Arthrospira platensis* is a species of planktonic photosynthetic cyanobacteria that thrives in large areas of biomass inside tropical and subtropical aquatic environments characterized by elevated levels of carbonate and bicarbonate salts, as well as an alkaline pH of 9 [46]. As an eco-friendly process, *Arthrospira platensis* can be cultivated on animal effluent (a low-cost medium) [47]. *Arthrospira* production in effluents from animal dung has many benefits, such as large cost savings and the resolution of waste disposal issues. Conditions such as temperature, nutrient levels, and salinity cause the composition of algal biomass to change [5,48,49]. Nutrient starvation closely changes the composition of biomass; nitrogen starvation leads to an increase in lipid accumulation [48], and phosphorus deficiency results in increased fat and carbohydrate levels [48].

*Arthrospira platensis* (synonym of *Spirulina platensis*) is one of the most nutrient-dense foods on the planet, and its use as a dietary supplement is increasing. It is gaining popularity as a nutritional supplement around the globe. It is rich in proteins, essential amino acids, polyunsaturated fatty acids, pigments, vitamins, and phenolics [36,50,51]. *Arthrospira platensis* has protein content ranging from 55 to 70% of its dry weight (dw), which is greater than that of egg (approximately 10% of its weight [52] and 17.0% in the yolk [53]) and meat (17–20% [54]) [36,55]. Proteins in milk and egg have a high digestibility rate of about 97% [56]. Secondly, meat, fish, and poultry have a high digestibility rate. Cyanobacteria such as *Arthrospira* also have a high digestibility rate of approximately 86% [57,58]. Knowing the definitions of some terms, such as digestibility, bioavailability, and bioaccessibility, helps us to understand the metabolism of nutrients. Digestibility refers to the amount of nutrients absorbed by an individual, and it is usually calculated by subtracting the amount of nutrients in the feces from the amount of food ingested [59].

*Arthrospira*, which also contains antioxidants, phytonutrients, probiotics, and nutraceuticals, is the most nutrient-dense, concentrated bacterium known in the diets of humans [20,36,60]. This cyanobacterium is not only recognized as one of the most valuable sources of protein, but also contains highly valuable fatty acids [linoleic (19–26%), gamma-linolenic (16–25%), oleic (3–8%), and palmitic (34–42%)]; vitamins (provitamin A, vitamin C, vitamin E, etc.); phenolic compounds; minerals such as iron, calcium, chromium, copper, magnesium, manganese, phosphorus, potassium, sodium, and zinc; and pigments (chlorophyll-a, phycocyanin, etc.) [27,36,60,61]. *Arthrospira* is also rich in the PUFAs—polyunsaturated fatty acids—as PUFAs account for 42–45% of total fatty acids [22]. These are crucial parts of a well-rounded diet that aid in nervous system development and help prevent or alleviate various diseases [20]. In Western diets, carotenoids play a vital role, accounting for roughly 30% of daily vitamin A consumption [36]. Zeaxanthin and β-carotene are all examples of carotenoids found in *Arthrospira* [62]. In a recent study, the dry weight content of *A. platensis* was reported to be 20.78 mg/100 g and 36.75 mg/100 g for carotenoids/zeaxanthin and β-carotene, respectively [62]. Due to its impressive nutrient makeup, which can be employed for therapeutic purposes, *Arthrospira* is quickly emerging as a comprehensive solution to diverse demands. The United Nations deemed *Arthrospira* to be the best food for the future at a food conference, and it is gaining popularity today [63]. Today, *Arthrospira* biomass is used to make nutritional supplements like dry powder, flakes, and capsules that are marketed as “superfoods” [20,36]. In a previous review, the effects of *Spirulina* supplementation (considering its antioxidant, immunomodulatory and anti-inflammatory effects) on pathological conditions in the population were examined by Calella et al. [64]. In this review, 18 studies consisting of 1621 records were evaluated. *Spirulina platensis* has been observed to be beneficial in both infectious and non-infectious patients. Improvement was observed in all cases (except male infertility). However, clinical studies about *Spirulina* are still very few. Therefore, many high-quality clinical studies are needed.

*Chlorella vulgaris* is a green microalga that might be exploited as a food source [19,20]. Aside from being offered in health food stores and as fish feed, *Chlorella* has become a popular supplement. *Chlorella* was considered a commercial microalga for use as a protein source (50–60% dw) [19,65]. The amino acid profile of a protein determines its nutritional quality. The essential amino acids produced by *C. vulgaris* biomass (Table 3) compare favorably and even exceed the conventional human nutrition profile recommended by the World Health Organization (WHO) and Food and Agricultural Organization (FAO) [21,24]. *C. vulgaris*, under optimal growth conditions, can reach a lipid content of 5–40% dw consisting of glycolipid waxes, phospholipids, and trace amounts of free fatty acids [21]. Diverse growth circumstances lead to changes in the composition of fatty acids (e.g., palmitic acid, stearic acid, palmitoleic acid, and oleic acid) that are suitable for different uses [24]. The β 1–3 glucan found in *C. vulgaris* is an essential polysaccharide with numerous beneficial effects on human health [24]. Additionally, it contains 9–18% dietary fiber, 1–2% chlorophyll, vitamins (like B1, B2, B3, B5, B6, B7, B9, B12, E, C, A, and vitamin K), and minerals (like Mg, K, Fe, and Ca). *Chlorella’s* most important commercial product is a series of by-products that are employed in the preservation of fruits and vegetables [66]. 

*Dunaliella salina* is a green halophilic microalga that is cultivated as a source of beta-carotene (up to 14% of its dry weight), glycerol, and photosynthetic pigment [30,67]. The orange pigment, β–carotene, is also used as a vitamin A supplement. Large-scale *D. salina* production may be found in both Australia and Israel; the commercial cultivation of this alga as a source of β-carotene dates back to the 1980s [30]. With a combined pond area of almost 900 hectares, the two Australian facilities are the world’s largest commercial microalgae production facility. For the pharmaceutical and nutraceutical industries, these plants generate “natural” β-carotene in the form of oil suspensions, beadlets, and water-soluble powder. Additionally, *D. salina* is harvested and dried and can be used as animal feed. Its algal biomass can be processed into a variety of useful substances such as glycerol, protein, enzymes, fatty acids, and vitamins. 

To increase β-carotene production from *D. salina*, the nutritional and environmental conditions in which the algae thrive can be changed [68]. Conditions such as salinity, irradiance, and nutrients alter the composition of *D. salina* biomass [68,69]. High salinity and irradiance stimulate β-carotene production in the halophilic microalga, which appears orange-red in masking due to increased β-carotene. Due to its provitamin and antioxidant activities, the US Food and Drug Administration (FDA) has classified *Dunaliella* as a food source that is Generally Regarded as Safe (GRAS), and it is primarily utilized for human and animal nutrition, food coloring, and cosmetics [20].

The microalga *Haematococcus pluvialis* is known for its capacity to collect high levels of astaxanthin. The annual biomass yield of *H. pluvialis* can reach over 300 tons [70], making it a popular choice in the biotechnology sector for the production of astaxanthin. The economic value of astaxanthin exceeds USD 240 million per year [71], with a market price of around USD 2000 per kilogram. Astaxanthin is a highly sought-after carotenoid [72]. In addition to neutralizing singlet oxygen, astaxanthin is an excellent scavenger of harmful free radicals [72,73]. The life cycle of *H. pluvialis* contains two distinct stages: the green motile stage and the red non-motile stage [74,75]. Unfavorable culture conditions like poor nutrients cause the vegetative motile green cells (macrozooids) to turn into red, non-motile hematocyst cells (aplanospores) [75]. In order to concentrate the biomass from the exhausted culture media, harvesting procedures are implemented once the non-motile hematocyst red cell has reached the mature state. Environmental variables such as culture medium, temperature, pH, and the amount of light are important factors for optimal growth conditions to achieve high cell density and astaxanthin accumulation in *H. pluvialis* [73,74,75]. Carotenogenesis is induced when cells are subjected to stressful circumstances caused by nutritional (nitrogen and phosphorus) deficiency, excessive salinity, and a combination of multiple stress factors that stimulate the accumulation of astaxanthin [73]. Depending on the conditions of cultivation, the production of astaxanthin in *H. pluvialis* might range from 3.8 to 5.0% dw [34,73,75,76].

### 1.3. Functional Food Products with Incorporation of Microalgae

Microalgae have been studied as a potential food source, especially a protein source for humans, since as early as the 1950s. The commercial cultivation of *Chlorella* and *Arthrospira* for protein supply began in the 1960s and 1970s, respectively [19,77]. The cultivation of *Dunaliella* and *Haematococcus* (especially β-carotene and astaxanthin) for food coloring was developed in the 1980s [19]. During the first decade of the twenty-first century, scientists started mass-producing polyunsaturated fatty acids, particularly omega-3. Due to their simple cultivation with high protein content and nutritional value, *Chlorella* and *Arthrospira platensis* are at the forefront of the microalgal market [63,78].

Due to their valuable chemical composition, microalgae have several commercial applications today, including (i) increasing the nutritional value of food and animal feed, (ii) playing an essential role in aquaculture, and (iii) the manufacturing of cosmetics [19,79,80]. Also, microalgae are farmed extensively as a source of very important biochemicals. As an example, PUFAs are added to infant formulas and dietary supplements, and pigments play a significant role as natural colors [19,20]. Three essential microalgal features can be transformed into technical and economic benefits. Microalgae are a very biodiverse group with a broad range of biochemical characteristics; as a result, they generate a variety of bioactive chemicals and unique lipids, proteins, essential amino acids, and carbohydrates [80,81,82].

Microalgae can be an extremely intriguing natural source of novel chemicals with biological activity that might be enable them to be exploited as functional components [19,36,83,84]. A variety of secondary (biologically active) metabolites are produced by some microalgal species that live in habitats subjected to heavy stress (such as changes in salt concentration and temperature, nutrient availability, or UV-V irradiation). As a result of their rapid adaptation to changing environmental conditions, these microalgal species have developed a wide range of unique secondary (biologically active) metabolites. Due to the taxonomic variety of microalgae, the hunt for novel physiologically active chemicals may be viewed as an almost limitless topic of study [30,73,85]. 

There are many different types of microalgae, but only a few are safe for human consumption. The FDA in the US awards the GRAS (Generally Recognized as Safe) classification to newly approved foods only after rigorous scientific testing has shown their safety. Several microalgae, such as *Arthrospira platensis*, *Chlorella vulgaris*, and *Dunaliella bardawil*, are examples of GRAS-approved microalgae [19,20,86]. The European Union’s Food and Feed Systems are regulated by the European Commission and the European Food Safety Authority. *Spirulina* and *Chlorella* are approved and the most widely consumed microalgae, and their entire biomass can be used in culinary products and marketed directly to consumers in European countries [19,20]. 

*Arthrospira* is the most healthy product known to humankind, according to the WHO. Moreover, *Arthrospira* is the most suitable food for the future, according to UNESCO. It is one of the main foods that can be grown on long-term space missions, according to NASA and the European Space Agency. The long history of *Arthrospira’s* use means that it can be commercialized in the European Union (EU) without having to comply with new food regulations [87]. The composition of *Arthrospira*, as well as the health benefits associated with consuming *Arthrospira* (or compounds derived from it), indicate that it has the potential to become a significant food and to be employed as an ingredient in the development of functional foods in the future [19,20,86]. It is “one of the greatest protein sources”, according to the FDA. *Arthrospira* from microalgae is allowed to be used by an intergovernmental organization to combat malnutrition [19,20].

Polyunsaturated fatty acids (PUFAs) found in microalgae (Table 4) have been shown to be effective in the prevention and treatment of a wide range of diseases, including cancer and cardiovascular disease [20,41,88]. PUFAs, particularly n-3 PUFAs such as α-linolenic acid (ALA, C18:3n-3), EPA (C20:5n-3), docosapentaenoic acid (DPA, C22:5n-3), and DHA (C22:6n-6), have been reported to be useful in preventing or treating numerous disorders (such as cancer, arthritis, cardiovascular diseases, asthma, type 2 diabetes, inflammatory bowel disorders, depression, and kidney and skin diseases) [19,20,89,90]. Considered nutraceuticals such as EPA and DHA have been extracted from several microalgal species farmed for this purpose [19,20,91]. According to the FDA, dietary sources of PUFAs such as EPA and DHA reduce the risk of coronary heart disease [19,20]. Microalgae are now farmed largely for the production of DHA, which is added to foods [92].

Cell wall polysaccharides differ among microalgal species [20,93]. Polysaccharides produced from marine microalgae are promising in many ways. This is because they are antioxidant, antiviral, and anticoagulant [94]. They are also much less toxic. Red microalgae such as *Porphyridium* sp. contain sulfated polysaccharides with anti-inflammatory properties [94]. Sulfated polysaccharides are the most thoroughly researched category of algal polysaccharides [20,95]. Sulfated polysaccharides obtained from *Arthrospira* also have an antiviral property [96,97]

**Figure 1:**
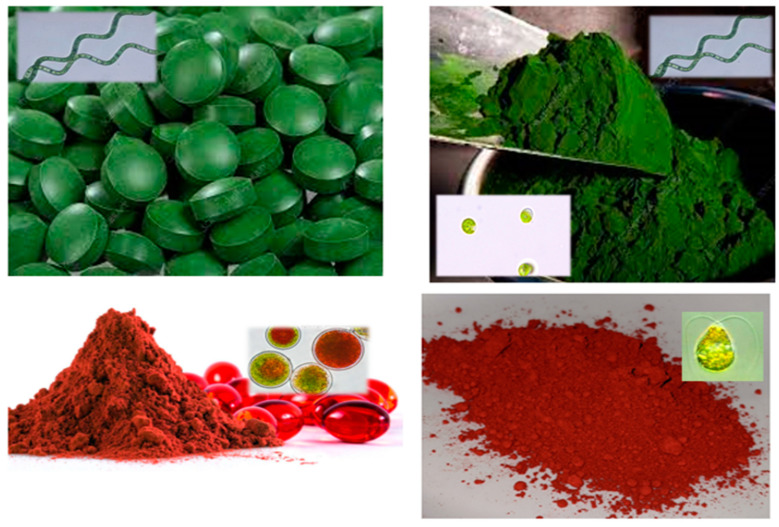
Microalgal commercial nutritional supplement (adapted from Priyadarshani and Rath [81]).

Bioactive chemicals, such as β-carotene (*D. salina*), astaxanthin (*H. pluvialis*), EPA, and DHA (*Chrypthecodinium cohnii*) [98], can be used in goods or taken as supplements [5]. As nutritional supplements, algal biomasses are supplied as pills, capsules, and liquids (Figure 1).

As biopeptides (protein hydrolysates) are more easily absorbed than proteins and amino acids, they are advantageous protein sources for humans [99]. High digestibility (83–90 percent) and all necessary amino acids (50–70 percent dry weight) are found in the biomass of *Arthrospira* [100]. It has been shown that biomass from microalgae is better for people in terms of nutrition and safety than traditional protein sources [21].

Microalgae are considered excellent sources of vitamins and antioxidants. Water-soluble vitamins and lipids are found in these organisms and can be used in food or as supplements. Folic acid, biotin, and vitamins are all present in microalgae [101]. Vitamin B12 and β-carotene (provitamin A) are found in *Arthrospira* [21]. *Arthrospira* consumption has been linked to an increase in gut *Lactobacillus* and improved dietary absorption of B1 and other vitamins [102]. *Arthrospira*, *Chlorella*, and *Dunaliella* species have been utilized to effectively create large quantities of important chemicals, including lipids, proteins, and pigments, as shown in Table 5 [103,104].

## 2. New Trends in Microalgae Food Application 

Fortified or enhanced foods have been produced since the turn of the 20th century, and they are foods whose natural composition has been modified by the addition of necessary nutrients. Micronutrient absorption and their use by the body are prerequisites for the fortification of foods to have a beneficial effect on nutritional status (bioavailability). Bioavailability is affected by nutritional status; the presence of substances in food that aid or hinder absorption; and interactions between micronutrients, diseases, and the chemical properties of the molecule used for fortification [105]. Iron deficiency anemia among children under the age of five has been significantly reduced in nations like Chile, Venezuela, and Mexico [106]. The salt iodization initiative has also demonstrated its efficacy in less than a decade [106]. Other programs have added zinc, vitamin A, and folic acid to diets because these nutrients are low in many populations, especially in newborns and children. According to the WHO, food fortification is a low-cost, relatively straightforward method that can reach a large audience and reduce the high incidence of micronutrient deficiencies that affect children in underdeveloped nations [107]. Losses in human capital have severe financial ramifications, as do their effects on health and the ability to innovate in the future.

Microalgal biotechnology has evolved and diversified tremendously during the past 30 years [108]. *Arthrospira*, for example, has been consumed by indigenous populations in Mexico and Africa for centuries. It was used to make tecuitlatl, a cake made with *Arthrospira* gathered from Lake Texcoco in Mexico [36]. *Arthrospira* from the alkaline Lake Kossorom was gathered in Chad and used to make a cake known as dihe [109]. Biomass has been used in many nutrient products to improve nutritional quality (Table 5) and to have a therapeutic effect on chronic diseases, so *Arthrospira* can also be used as a functional ingredient [110,111].

Consumers’ concerns about the health and safety of eating processed foods have grown in recent years. As a result of an increased risk of cancer or allergic reactions, the FDA and other national agencies have limited the use of synthetic dyes. As a result, natural additives will be increasingly popular in the food sector [112], and microalgae might play a role in this development. *Chlorella*, *Dunaliella*, and *Arthrospira* are only a handful of the many microalgae genera that are commercially accessible for human nutrition [63].

Several species have been commercially farmed, and the resulting biomass has been utilized to make food-grade goods. They are used to supplement natural meals [113]. Microalgae have not only been used in pill capsule, tablet, and powder form, but also added to foods (pasta, snack foods, and beverages) either as dietary supplements or as natural food dyes [78,114]. They has been produced as a functional food oil, abundant in fatty acids and antioxidants, and tinted with carotenoids derived from microalgae. In addition, heat application (like cooking) did not cause any loss in micronutrients in cooked foods, such as pasta, bread, and cookies enriched with microalgae [115,116]. On the contrary, lower cooking loss and a higher swelling index were obtained in cooked microalgae-containing foods compared with control samples [115]. A significant increase in pasta hardness was reported with an increase in added microalgae due to structural reinforcement [115]. 

**Figure 2:**
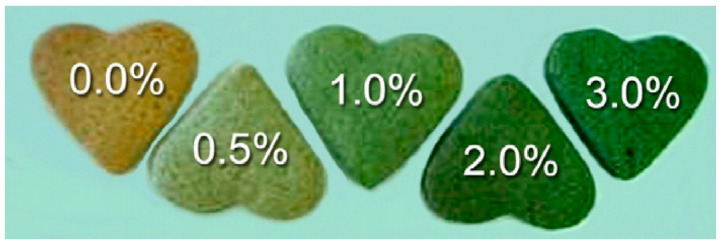
Cookies with different incorporation levels (0.0, 0.5, 1.0, 2.0, and 3.0%) of *Chlorella vulgaris* biomass [120].

*Chlorella vulgaris* is sold as a food supplement, an additive [117,118], a food color, and an emulsion for food products [119]. The textural features of the biscuits were improved, and the color and texture were stable for three months, as previously reported for *Chlorella* biscuits [120]. When the biomass content was increased from 1.0 percent to 3.0 percent, the biscuits changed from a brownish to a green and duller tone (Figure 2).

**Figure 3:**
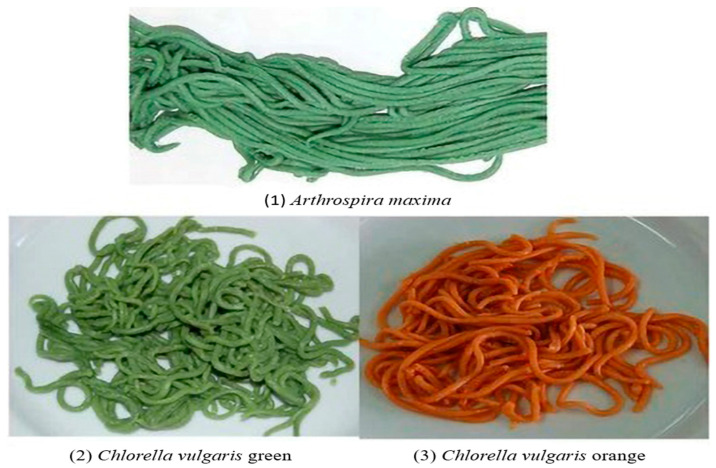
Pasta incorporated with (**1**) *Arthrospira maxima*, (**2**) *Chlorella vulgaris*, and (**3**) *Chlorella vulgaris* orange [118].

*Arthrospira platensis* has been utilized to develop functional food products because it contains proteins, unsaturated fats, the B vitamin group, several minerals, and phycocyanin [19,36,121]. In more recent investigations, the incorporation of microalgal biomass into food items has been explored to increase their nutritional characteristics. Using *Chlorella vulgaris* and *Arthrospira maxima* microalgal biomass, Fradique et al. [118] created products with improved chemical content without compromising baking quality (Figure 3).

**Figure 4:**
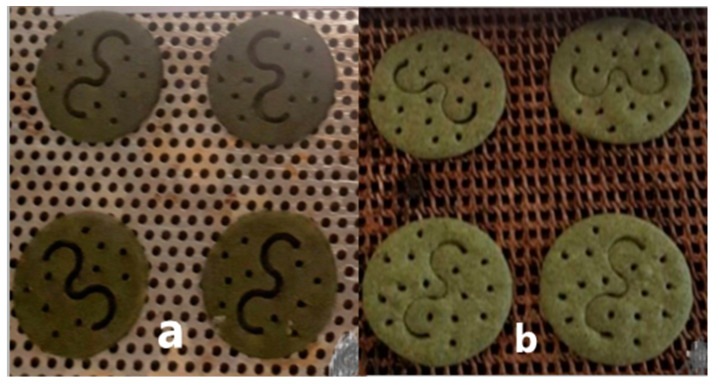
Functional biscuits with 4% *A. platensis* and different amounts of oil, sugar, and flour between (**a**,**b**) [27].

A study was conducted to enhance the characteristics of biscuits through the incorporation of *A. platensis* [27]. The optimal outcomes from a series of 30 distinct biscuit manufacture experiments are presented in Figure 4. The incorporation of *A. platensis* (4%) in the biscuit formulation resulted in an enhancement in its flavor, as evidenced by an increase in hardness and crispness levels (Figure 4). The incorporation of 4% *A. platensis* resulted in a notable enhancement in both the protein content (57%) and the amino acid content of the biscuit. It was discovered that there is significant potential for enhancing the nutritional composition of the biscuit.

**Figure 5:**
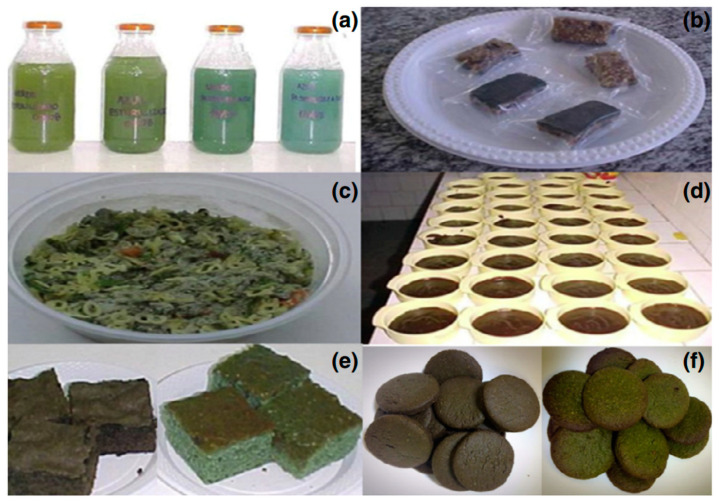
Food enriched with *Arthrospira* sp.: (**a**) isotonic beverages, (**b**) cereal bars, (**c**) instant soups, (**d**) pudding, (**e**) cake powder mix, and (**f**) biscuits [123].

Malnourished persons can be supported with *Arthrospira*-containing functional foods such as chocolate, biscuits, and others [122]. The physical, chemical, and sensory features of the chocolate cookies enriched with *A. platensis* were investigated, as well as the digestibility of the product. The protein level of the diet with the addition of 5% algal biomass exhibited a protein content greater than the control. Biscuits enriched with *Arthrospira platensis* were 86% more digestible than other cookies containing microalgae and more popular with the judges compared with other cookies incorporating the microalgae [122]. Various foods (Figure 5) have been produced in a biochemical engineering laboratory with the addition of *A. platensis* by the *Spirulina* Food Enrichment Center of the Federal University of Rio Grande [123].

The effect of adding *Arthrospira platensis* (0–1% concentrations) on the growth of microflora and the physicochemical properties of ayran before and after fermentation and on the 7th, 14th, and 21st days of storage was evaluated [124]. *A. platensis* at 1% had the highest total solid and protein content. *Arthrospira platensis* has the potential for boosting the growth of probiotic bacteria and the nutritional value of ayran [124]. *Arthrospira platensis* biomass, whey protein hydrolysates, and probiotics were used to develop functional ayran [125]. They have boosting effects on the growth of microflora and the nutritional value of ayran. They offer significant promise for increasing the nutritional content of ayran and the development of probiotic cultures [125].

**Figure 6:**
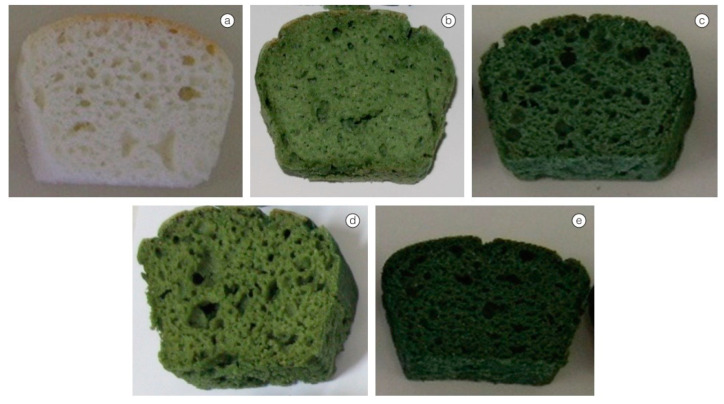
Gluten-free bread produced using rice flour: (**a**) control and with addition of (**b**) 2%, (**c**) 3%, (**d**) 4%, and (**e**) 5% *Arthrospira platensis* [126].

Products using *A. platensis* and rice flour (a substitute for wheat flour) to provide gluten-free bread to people with celiac syndrome are given in Figure 6 [126]. Greater protein content was detected in gluten-free loaves made from rice flour with the addition of 2% to 5% *A. platensis* [126]. The results indicated that the protein content increased by 39.04% in the bread when the microalgal biomass was increased to 5.0%. Microalgae also improved the amino acid composition, with substantial increases in 11 amino acids (four of which are important, such as threonine, methionine, isoleucine, and leucine), when compared to the control group without microalgae. Gluten-free breads with 5.0 percent microalgae biomass added had the same preference as those with 3.0 percent. Adding *A. platensis* at various concentrations can increase protein, total fat, and mineral content in foods [127]. At the same time, the results of the sensory tests of these formulated cakes were reported to be positive.

**Figure 7:**
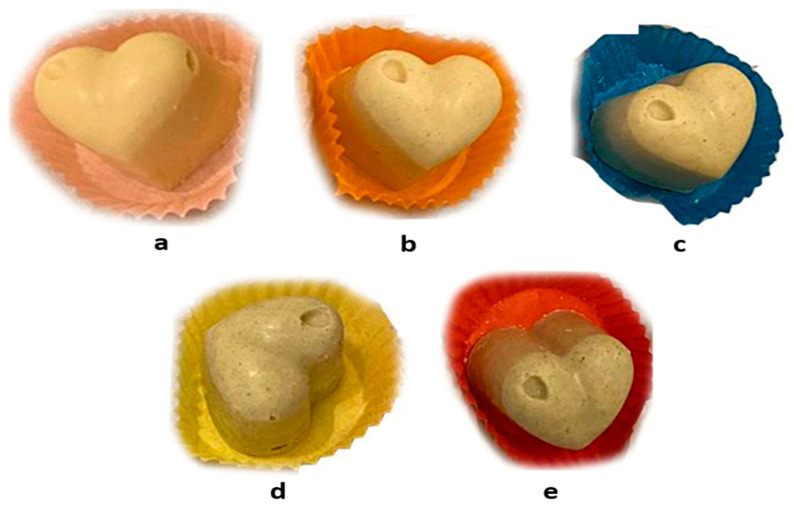
White chocolate samples: (**a**) control, and (**b**) 0.5%, (**c**) 1%, (**d**) 2%, and (**e**) 4% *Arthrospira*-containing samples [128].

The incorporation of *Arthrospira platensis* (0.0–4.0%) to enhance the nutritional and sensory attributes of white chocolate is given in Figure 7 [128]. Since there is no cocoa content in white chocolate, it was found to be deficient in antioxidants, so the chocolate content was enriched with the addition of *A. platensis* [128]. The addition of 4% *A. platensis* increased the product’s protein content by 23.1%, its total amino acid content by about 45%, its lipid content by 10.3%, and its mineral content by 13.5%. The addition of 4% *Arthrospira* to white chocolate significantly increased the overall fat content (especially linoleic acid). The total PUFA content increased significantly by 45% in the microalgae-enriched (4%) sample. In particular, it showed a significant increase in iron content (from 9.20 mg/kg to 93.13 mg/kg in the sample containing 4% *Arthrospira*). *Arthrospira platensis* had a boosting effect on the iron content of the chocolate [128].

**Figure 8:**
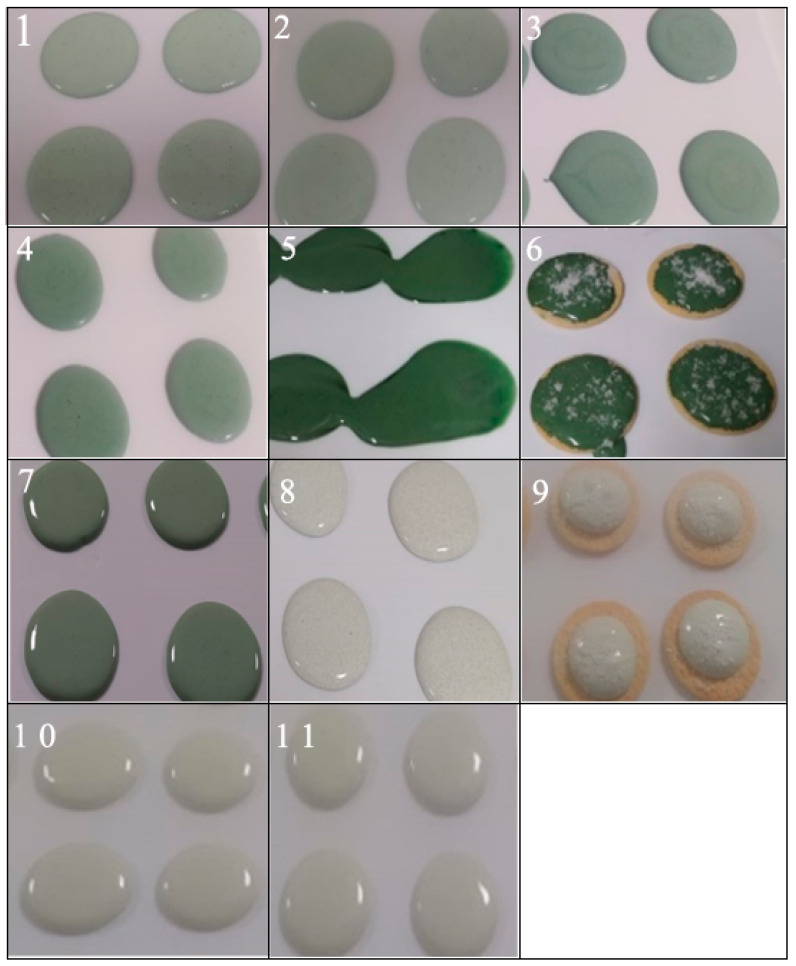
Applications of *A. platensis* biomass as a substitute and gelling agent in marshmallow production. *Arthrospira platensis* levels: 0.6% in trials 1, 2, and 9; 1.2% in trials 3, 4, 7, 8, and 10; and 1.8% in trials 5, 6, and 11. Temperature: 30 °C in trials 1–6; 60 °C in trial 7, and 80 °C in trials 8–11 [129].

Çelekli and Maraşlı [129] sought to shed light on the potential applications of *A. platensis* biomass as a substitute, herbal innovation, and gelling agent in aerated and foamy confectionery and to assess the effects of gelatin substitution rate, the hydration (dissolution) temperature of an alternative stabilizer, and aeration temperature in marshmallow production. In the use of *A. platensis* biomass in food, both the denaturation and gelation behavior of the protein fraction and also the interactions of the pigments and proteins in the composition should be taken into account. *Arthrospira platensis* protein isolates are obtained by a two-stage process with heat induction. These steps are (i) protein chain unwinding and (ii) aggregation. The first stage is reversible, while the second stage is not. In addition, when protein isolates of *A. platensis* are heated to a temperature above the denaturation temperature and recooled to ambient temperature, gelation is inevitable and does not pose a disadvantage based on this confectionery technology. This process was used to develop a novel marshmallow product (Figure 8) [129]. This gelling behavior, in which hydrophobic interactions are important, and the effect of intermolecular disulfide bonds on gel formation is low but important in terms of the physical properties of this gel, is still an area that can be defined as complex and needs further studies.

## 3. Challenges in Food Products with Microalgae

**Figure 9:**
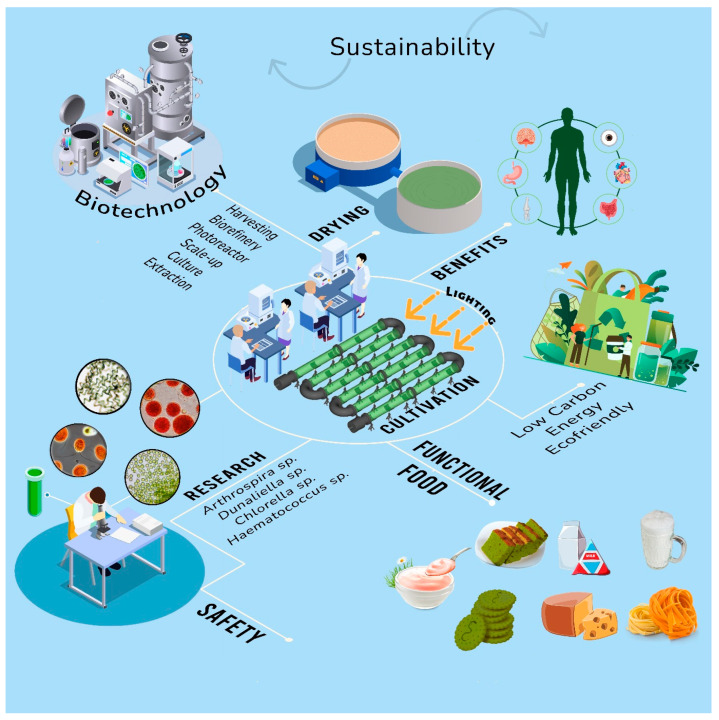
The potential for future food production using microalgae.

Food and nutrition security and a healthy lifestyle for present and future generations require novel and sustainable food production techniques with low environmental implications (Figure 9). It is also important that it is not out of reach financially, is easy to get to, and is accepted by the local culture. People who are health- and nutrition-conscious, as well as those who are concerned with sustainability and eco-friendliness, would be inclined to eat goods containing microalgae. The same justifications would hold true for sensory attributes such as taste and smell. To improve product quality and develop the market, it is important for us to understand consumer attitudes about food products made from microalgae. By doing so, we can pinpoint our target market and meet their needs.

The use of microalgae in functional foods has several disadvantages, such as its cost, its flavor, the loss of some bioactive compounds during processing, consumer reflex, its intensive color, etc. While there has been constant growth in the demand for higher-value microalgae-based goods in recent years, there are still challenges that must be addressed before these industries can reach their full potential. The economic sustainability of the commercial-scale production of microalgae biomass is still in the shadow of doubt, especially the cultivation and harvesting process. Costs associated with production, harvesting, and processing have been extensively discussed in prior studies and are a major factor preventing the widespread use of this technology [130]. These fundamental recommendations may provide a useful road map for efficient and inexpensive algal biomass production. The harvesting procedure is one of the more expensive parts of microalgal production, typically accounting for 20–30% of the overall cost [131,132]. Currently, 90% of the cost of obtaining microalgal biomass from open ponds is attributable to energy-intensive harvesting techniques such as flotation, centrifugation, filtration, and electricity-based techniques [132,133]. Finally, a peek at future biotechnologies that will combine to generate, harvest, and process microalgae utilizing eco-friendly and cost-effective approaches is proposed.

Harvested *Arthrospira* are typically dried to make their storage and inclusion in food more convenient. The drying techniques (e.g., solar drying, air drying, vacuum drying, free drying, and spray drying) are another key challenge that has a substantial impact on the dried microalgal biomass [86]. Drying techniques can severely deplete the nutrients of microalgae. When comparing the protein content of spray-dried *Arthrospira* to that of newly harvested biomass, a loss of 10–25% was found. Likely, freeze drying resulted in a roughly 10% decrease in protein content [134]. Conventional drying also caused a loss of phycocyanin, ranging from 50% to 90% in *Arthrospira* biomass [135,136]. Additionally, the dried *Arthrospira* biomass included significantly lower values of bioactive compounds than those of freshly harvested biomass [86,136]. Furthermore, the costs associated with drying *Arthrospira* amount to about 30% of the entire production price. The company Cyanotech Corporation (Kailua-Kona, HI, USA) has used the Ocean Chill^TM^ drying method to produce Hawaiian *Spirulina* pacifica in a BioSecure Zone free from pollution. The company asserted that the Hawaiian *Spirulina* pacifica included a greater concentration of nutrients. Recently, drying using infrared radiation combined with microwave drying has been used as an innovative approach [137].

### 3.1. Challenges for Sensory Qualities of Food in Food Products with Microalgae

One of the major problems that has adversely affected the microalgae sector is the undesirable sensory properties of microalgae [5]. Products such as powders, tablets, and beverages from dried *Arthrospira* had a smell or fishy taste [21]. When fresh *Arthrospira* is added to food or drink, it barely alters the smell and flavor. The integration of microalgae that has not undergone component extraction imparts an unpleasant flavor above a certain concentration, rendering the food undesirable to the majority of customers, especially those who have never consumed algal-based goods. Upon the addition of *A. platensis* and *C. vulgaris* into yogurt, the results of a sensory evaluation indicated a more unpleasant flavor of *A. platensis* compared with *C. vulgaris* [138]. This inappropriate flavor is caused by the oxidation of polyunsaturated fatty acids and other compounds from microalgae. Adding different amounts of *Arthrospira* to yogurt (0.25, 0.5, 0.75, and 1%) changes the fermentation process, texture, and nutritional or sensory qualities of the products [139]. The textural qualities and sensory acceptability of the final milk product were preserved when 0.25% *Arthrospira* was incorporated into the yogurt. According to research by Gyenis et al. [140], fermented milk with 3 g/dm3 of microalgal biomass was the best option in terms of sensory qualities and price [141].

When the addition of microalgae to food products is lowered to decrease the disagreeable taste and odor, this leads to a decrease in protein content and other bioactive substances in food products. In terms of food type, it is easier to include microalgae in baked items such as bread, cookies, and pasta than other dietary items, such as yogurt [19,20]. Batista et al. [142] reported that cookies made with 2% (*w*/*w*) *Arthrospira* achieved positive results regarding their flavor.

Various sources of proteins (e.g., *Spirulina*, *Chlorella*, pea, lentil, and broad bean) were added into turkey Burgers to evaluate the physicochemical characteristics, textural attributes, and nutritional value of meat products [143]. The maximum values of amino acids correspond to turkey burgers formed with *Spirulina* and broad bean proteins. Glutamic acid was the predominant one, obtaining a value of 2.13 g/100 g in the case of broad *Spirulina* protein [144].

One of the primary obstacles preventing the widespread use of microalgal dry biomass in the food industry is the intense color created by microalgae. As a result of the presence of microalgae, the color of food can be altered, which may not be acceptable to most people, and the quality of commonly consumed foods like bread and dairy products is affected. Nonetheless, it serves a useful purpose for a few other items, such as pasta, since people eat it in a wide variety of colors. An innovative strategy to cover up the taste of microalgae is spray-drying microencapsulation. The unique taste of microalgae was shown to be effectively hidden when octenyl succinic anhydride starch was used as a coating material. It was found that adding microencapsulated *Arthrospira* to wheat cookies at a 20% (*w*/*w*) concentration did not significantly alter either purchase intent or overall acceptance compared to the control group. When 20% (*w*/*w*) *Arthrospira* was added to the biscuits, the protein content increased by 40%, and the ash content increased by 70%, which were significantly higher than in previous works of a similar nature [145].

There are also new ways to handle the bright color of microalgae. The European Food Safety Authority recently approved two pale-colored *Chlorella* powder products with low chlorophyll content as food raw materials and food supplements [146]. These newest items are more aesthetically neutral and consumer-acceptable than conventional dark green products. Additionally, the C/N ratio of the cultivation medium leads to a change in the color of microalgal biomass [147,148]. Microalgae are viewed as a sustainable, environmentally friendly, and nutritious health product by Spanish consumers, according to a recent evaluation of consumer knowledge and attitudes conducted by Lafarga et al. [36]. However, a lack of information on the product and a lack of consumption habits were barriers to the active consumption of this product. The most appealing aspect of microalgae to consumers is its health advantages. Numerous clinical investigations have established the obvious health benefits of microalgae as nutritional supplements. Therefore, the current scant research shows that educating consumers about microalgae can raise demand for and sales of products that include these microorganisms.

Despite the fact that cultural influences and future opportunities to mainly investigate customers’ views for adopting new eating habits that involve more novel food items may lead perceptions and attitudes towards food to differ, the results described here might be generalized to other European countries.

Consumers’ ability to tolerate the taste of microalgae-based foods is one such factor [19,20]. Key factors in the development of the microalgae-based foods market will include the type of microalgae used, the method used to prepare the microalgal biomass (i.e., as a dried powder or by processing), the combination of ingredients, and the shape of the final product. Among the many issues with using microalgae in food is their pungent smell and fishy flavor. Participants took a designed test to assess their views toward unfamiliar foods, since food neophobia can alter how well-liked novel foods are [19,20]. Consumers believed that the product was better for their health, and because of this, they were willing to pay a higher price for it. This information may prove helpful in the process of promoting and selling this unique product.

Products containing microalgae would find an interesting consumer base among those who are health-conscious, nutrition-conscious, or concerned with environmental issues and sustainability. Similar arguments can be made in terms of other senses, such as smell and taste. To better meet the needs of our target market and expand our business, we need to have a firm grasp on how microalgae-based food products are perceived by those who consume them.

In addition to problems with customer acceptability and a lack of production capacity, the market for foods made from microalgae has problems with legislation and regulation. Laws and rules governing the consumption of algae differ from country to country.

Some of the obstacles that prevent the widespread adoption of a food or ingredient generated from a “new” organism include customer acceptance, ambiguous regulatory frameworks, and the reluctance of investors to provide financial and commercial backing for these inventions. If academics want their work to be successfully translated into the commercial sector, they must have a firm grasp of these issues. Only a small number of microalgal taxa are currently recognized on a global scale.

Incorporating microalgae into food could also change how bioactive substances in microalgae are absorbed and used by the body. So, there needs to be a full look at the health benefits of foods made from microalgae. This will make it easier to market foods made from microalgae as “functional foods”. Also, the type of product can affect how well consumers accept microalgae and how much is added, so it is important to promote foods made with microalgae.

We must assess the current status of microalgae-based products on the market and those still undergoing research and development, as well as gain a deeper understanding of the constraints and obstacles that can limit the use of microalgae as food ingredients. Considering all these obstacles, it is commonly acknowledged that conventional food production methods are inadequate to meet global food demand; production processes must be modified to be more sustainable and scalable.

### 3.2. Food Safety and Potential Risks

There are three factors that affect the safety of algae foods in relation to the algae. These may be physical and chemical pollutions and microbiological contaminations [149]. These factors endanger food safety. Therefore, new technologies are needed to detect these contaminants or pollutants quickly. There are now many new developments in the monitoring of heavy metals, algal toxins, and other contaminants. In the future, not only will there be cheap, fast, and safe detection methods for assessing algal food contamination, but these methods will also be linked to new technologies that work with artificial intelligence, biosensors, and molecular biology.

Foods enriched with either microalgal biomass, microalgal supplements, or biochemical compounds extracted from microalgae are subject to the same regulations that apply to all foods. These products must comply with food regulations, such as the requirement for food to be free from contamination or solvent residues. For example, solvent residues may be present in the production of fatty acids produced from microalgae [150], but this is not permitted by the authorities.

### 3.3. Challenge in Ensuring the Stability of the Nutritional Content of Microalgae

The biochemical content of microalgae is affected by light, humidity, pH, and high temperature, i.e., they are quite unstable [151]. They have a high tendency to deteriorate. It is important to ensure the stability of the biochemical component of microalgae. The important thing is to determine how we can take these ingredients at the highest rate for bioavailability.

To deal with the poor physicochemical stability of microalgae bioactive extracts, especially carotenoid, astaxanthin, and free fatty acids, numerous studies have suggested the coating of these bioactive compounds with biopolymer layer. This can significantly increase the stability of bioactive compounds under different conditions [152]. Encapsulation can ensure bioavailability and stability. Encapsulation generally refers to the process of incorporating a specific ingredient into a matrix, while an “encapsulation system” generally refers to a system designed to encapsulate, protect, and release the target active compounds [153].

## 4. Conclusions

Microalgae have great potential to serve as a valuable source of useful bioactive compounds for functional food supplements, nutraceuticals, cosmetics, and pharmaceuticals. It is widely acknowledged that the relationship between diet and disease is the cornerstone on preventive nutrition. Also, microalgae have received remarkable attention for the development of multifunctional food products that possess the potential to enhance human health. The production of functional foods containing bioactive components from microalgae enhances long-term possibilities for sustainable development. Microalgae such as *Arthrospira platensis* and *Chlorella vulgaris* have been highlighted as natural sources of protein, whereas *Dunaliella salina* and *Haematococcus pluvialis* are considered sources of pigment (especially because of their content of β-carotene and astaxanthin, respectively). Microalgae are a very biodiverse group with a broad range of biochemical characteristics; as a result, they generate a variety of bioactive chemicals and unique lipids, proteins (essential amino acids), and carbohydrates. Chemicals like β-carotene (*D. salina*), astaxanthin (*H. pluvialis*), EPA, and DHA (*Chrypthecodinium cohnii*), and others, can be obtained from various microalgae species and have been shown to be used as supplements in products or as dietary additions.

To improve product quality and develop the market, it is important for us to understand consumer attitudes toward food products made from microalgae. By doing so, we can pinpoint our target market and meet their needs.

The inclusion of microalgae in food may also change how the bioactive substances in microalgae are absorbed and used by the body. Therefore, it is necessary to fully explain the health benefits of foods made from microalgae. This would make it easier to market foods made from microalgae as “functional foods”, because consumers believe these products are better for their health, and because of this, they are willing to pay a higher price for it. This information may prove helpful in the process of promoting and selling this unique product.

## Figures and Tables

**Table 1:** Categories of functional foods.

Category	Example
Basic	Carrots (containing the anti-oxidant β-carotene)
Processed foods	Oat bran cereal
Processed foods with added ingredients	Calcium-enriched fruit juice
Food enhanced to have more of a functional component	Tomatoes with a higher level of lycopene
Isolated, purified preparation of active food ingredients	Isoflavones from soy β-glucan from oat bran

**Table 2:** Biomass composition of microalgae as macronutrient based on dry weight.

Algae	Protein	Carbohydrates	Lipid	References
*Chlorella vulgaris*	42–58	12–17	10–22	[23,24,25,26]
*Arthrospira platensis*	45–70	8–25	4–12	[21,22,27,28,29]
*Dunaliella salina*	38–57	4–6	6–31	[21,30]
*Haematococcus pluvialis*	5–45	36–40	25–37	[31,32,33,34]

**Table 3:** Composition of essential amino acids (g/100 g) in conventional and microalgal sources (dry weight).

Amino Acid	*Chlorella* sp.	*Arthrospira* sp.	*Dunaliella* sp.	*Haematococcus* sp.	Soy Bean
Lysine	8.4–8.9	4.6–4.8	2.4–2.7	1.4	6.4
Leucine	8.8–9.2	8.0–9.8	3.9–5.7	2.6	7.7
Isoleucine	3.8–6.7	6.0–6.7	1.9–2.8	1.1	5.3
Threonine	4.7–4.8	4.6–6.2	1.5–2.8	1.9	4.0
Methionine	2.2	1.4–2.5	0.8–1.0	-	1.3
Phenylalanine	5	4.9–5.3	2.5–2.8	1.5	5.0
Valine	5.5–6.1	6.5–7.1	2.0–2.9	1.5	5.3
Arginine	6.4	7.3	3.0–7.3	2.1	7.4
Histidine	2.0	2.2	0.8–1.8	0.6	2.6
Tryptophan	2.1	5.3	0.7–1.4	-	1.4
Reference	[19,21,26]	[19,21,28]	[21,37]	[38]	[21]

**Table 4:** Fatty acid composition (%) in microalgae.

	*H. pluvialis*	*H. pluvialis*	*C. vulgaris*	*D. salina*	*D. salina*	*A. platensis*	*A. platensis*	*A. platensis*
Palmitic acid (C16:0)	4.38	28.70	17.2	12.16	21.53	45.92	37.6	44.9
Oleic acid (C18:1n9c)	16.11	0.95	11.7	-	52.18	-	1.5	-
Linoleic acid (C18:2n6c)	7.04	2.47	-	-	12.42	-	-	-
γ-linolenic acid (C18:3n6)	4.30	3.06	-	-	-	-	-	-
α-linolenic (C18:3n3)	21.20	3.06	-	-	2.82	-	-	-
PUFA	51.51	-	21.9	-	15.50	-	19.4	-
MUFA	17.43	50.07	35.2	-	53.66	-	8.3	-
SFA	31.06		26.7	19.75	-	-	67.4	-
Caproic (C6:0)	18.23	-	-	-	-	-	-	-
Caprylic (C8:0)	3.20	0.04	-	0.54	-	-	-	-
Myristic (C14:0)	3.10	1.79	1.1	1.29	0.75	-	1.0	0.8
Palmitoleic (C16:1)	0.33	-	-	2.88	1.41	2.74	-	2.3
Heptadecenoic (C17:1)	0.97	-	-	-	0.08	-	-	-
Stearic (C18:0)	2.16	17.25	3.0	3.64	8.46	0.89	1.0	2.2
Arachidonic (C20:4n6)	4.79	0.27	-	-	0.26	-	-	-
Lignoceric (C24:0)	nd	0.00	-	-	-	-	-	-
C20:2	6.98	0.01	-	-	-	-	-	-
C20:5n3	-	0.01	0.0	-	-	-	0.0	-
Reference	[39]	[40]	[41]	[42]	[43]	[44]	[44]	[45]

**Table 5:** Main bioactive compounds extracted from microalgae [82].

Microalgae	Bioactive Compounds
*Arthrospira* species	Polysaccharides, phycocyanin, C-phycocyanin, allophycocyanin, phenolic acids, tocopherols (vitamin E), neophytadiene, phytol, PUFA (*n*-3) fatty acids, oleic acid, linolenic acid, palmitoleic acid, diacylglycerols, terpenoids, alkaloids, flavonoids
*Chlorella* species	Carotenoids, sulfated polysaccharides, sterols, PUFA (*n*-3) fatty acids, canthaxanthin, astaxanthin, peptide, oleic acid, eicosapentaenoic acid (EPA), zeaxanthin, violaxanthin, lutein, phenolic, terpenoids, alkaloids, phytol, phenol
*Haematococcus pluvialis*	Astaxanthin, lutein, zeaxanthin, canthaxanthin, lutein, β-carotene, oleic acid
*Dunaliella salina*	All-*trans*-β-carotene, all-*trans*-zeaxanthin, all-*trans*-lutein, *cis*-betacarotene, β-carotene, oleic acid, linolenic acid, palmitic acid, diacylglycerols, sterols

## Data Availability

The original contributions presented in the study are included in the article, further inquiries can be directed to the corresponding author.
